# Adoption of workplaces and reach of employees for a multi-faceted intervention targeting low back pain among nurses’ aides

**DOI:** 10.1186/1471-2288-14-60

**Published:** 2014-05-01

**Authors:** Charlotte Diana Nørregaard Rasmussen, Anne Konring Larsen, Andreas Holtermann, Karen Søgaard, Marie Birk Jørgensen

**Affiliations:** 1 National Research Centre for the Working Environment, Lersø Parkallé 105, 2100 Copenhagen Ø, Denmark; 2 Institute of Sports Science and Clinical Biomechanics, University of Southern Denmark, Campusvej 55, 5230 Odense M, Denmark

**Keywords:** RE-AIM, Participation, External validity, Health care workers, Musculoskeletal disorders

## Abstract

**Background:**

Workplace adoption and reach of health promotion are important, but generally poorly reported. The aim of this study is therefore to evaluate the adoption of workplaces (organizational level) and reach of employees (individual level) of a multi-faceted workplace health promotion and work environment intervention targeting low back pain among nurses’ aides in elderly care.

**Methods:**

Percentage of adopters was calculated among eligible workplaces and differences between adopters and non-adopters were evaluated through workplace registrations and manager questionnaires from all eligible workplaces. From the adopted workplaces reach was calculated among eligible employees as the percentage who responded on a questionnaire. Responders were compared with non-responders using data from company registrations. Among responders, comparisons based on questionnaire data were performed between those consenting to participate in the intervention (consenters) and those not consenting to participate in the intervention (non-consenters). Comparisons were done using Student's t-test for the continuous variables, Fisher's exact test for dichotomous variables and the Pearson’s chi^2^ for categorical variables. Moreover odds ratios for non-responding and non-consenting were investigated with binary logistic regression analyses.

**Results:**

The project was adopted by 44% of the offered workplaces. The main differences between adopters and non-adopters were that workplaces adopting the intervention had a more stable organization as well as a management with positive beliefs of the intervention’s potential benefits. Of eligible employees, 71% responded on the questionnaire and 57% consented to participate. Non-responders and non-consenters did not differ from the responders and consenters on demographic factors and health. However, more non-responders and non-consenters were low skilled, worked less than 30 hours pr. week, and worked evening and nightshift compared to responders and consenters, respectively. Consenters had more musculoskeletal pain and reduced self-rated health, as well as higher physical exertion during work compared to non-consenters.

**Conclusions:**

Our recruitment effort yielded a population of consenters that was representative of the target population of nurses’ aides with respect to demographic factors, and health. Moreover more consenters had problems like pain and high physical exertion during work, which fitted the scope of the intervention.

**Trial registration:**

The study is registered as ISRCTN78113519.

## Background

Non-communicable diseases are a main contributor to disability and mortality in the Western world [1], and initiatives are therefore applied in numerous settings with the aim of preserving, improving or reducing mutable health problems effectively [2]. The workplace has been pointed out as a relevant setting for health promotion [3]. However, workplace interventions that failed to be effective are well represented in the scientific literature [4]. For an initiative to be effective, it is important that it is feasible – meaning that it is attractive for workplaces (that workplaces are willing to adopt the initiative) and that it is effectively implemented among all or most of the employees (that employees are willing to participate in the initiative). A review suggests that the lack of effectiveness is due to poor participation from workplaces and employees [5]. However, workplace adoption and reach of employees are generally poorly reported in scientific literature [5]. Therefore, more knowledge of workplace adoption and reach of employees to enhance future intervention effectiveness is needed.

Adoption is defined as the absolute number, proportion, and representativeness of settings that are willing to introduce an initiative [6,7]. The adoption of health promotion initiatives has been reported in national registry studies [8], but is seldom reported in experimental trials [6,9]. This is despite the fact, that adoption constitutes important knowledge about a trial’s external validity. Furthermore, characteristics of adopting and non-adopting workplaces may help understand the dissemination potential of an initiative. For example, it is stated that workplaces that are innovative and have an effective leadership are more likely to adopt a new project [10]. Moreover workplace size, management-employee relations, management beliefs about the benefits of health promotion, as well as previous history of health promotion at the workplace may influence workplaces willingness to introduce an initiative [8]. However, the importance of these factors on workplace adoption needs further investigations.

Reach is defined as the percentage and representativeness of the participants willing to participate in an initiative [7]. In experimental trials, reach is more frequently reported than adoption [6] . However, information to allow for assessment of the representativeness of those who participate in trials tends to be reported less frequently than just reporting the percentage of the participants [6]. The participation levels in workplace health promotion are typically below 50% [5]. Low participation in interventions may have important consequences for the effectiveness (meaning that studies show no effect) and may also raise concerns about the external validity of the results. Moreover, if non-participants systematically differ from participants there is a risk of selection bias and low external validity [11]. Low participation and selection bias both minimize the potential public health impact of the interventions delivered [12]. Therefore the characteristics of participants as well as non-participants at both workplace and the employee level need to be reported. Information about characteristics of participants as well as non-participants have clear implications for the feasibility of the trial, the representativeness of the population, and consequently the external validity and the generalizability of the results [13].

We designed a multi-faceted intervention to prevent and reduce low back pain (LBP) and consequences of LBP among nurses’ aides in elderly care in Denmark. The study was registered with a unique trial registration number: ISRCTN78113519. Details regarding the overall concept and design of the trial are described in a previous publication [14]. The intervention is time-consuming, but flexible to adjust to different workplace settings and is built on a comprehensive theoretical framework of both effectiveness and implementation. Therefore, it is highly relevant to investigate the trial’s dissemination potential in terms of adoption among eligible workplaces and its implementation potential in terms of reach among eligible employees. Adoption and reach constitute two important measures of a process evaluation, and the concepts for the current study are inspired by the RE-AIM framework (Reach, Effectiveness, Adoption, Implementation, and Maintenance) [13]. The RE-AIM framework was designed to assess the external validity of an intervention as well as the potential for sustainable implementation and public health impact in real-world settings [13]. Therefore, the aim of this study is to evaluate the reach and adoption of a multi-faceted workplace health promotion and work environment intervention among nurses’ aides in elderly care in Denmark.

## Methods

The study is a cross-sectional evaluation of the reach and adoption of a multi-faceted cluster randomized workplace intervention among nurses’ aides in elderly care in Denmark aiming at preventing and reducing LBP and consequences of LBP. In short, the intervention lasts three months and consists of a combination of participatory ergonomics, physical exercise and cognitive behavioral therapy tailored to the target group. Moreover the intervention is built on a comprehensive theoretical framework of both effectiveness and implementation, e.g. the use of intervention mapping [15] in developing and planning of the intervention and the use of a participatory approach. The intervention will be conducted during paid working hours and will be supervised by local trained physiotherapists and occupational therapists. The intervention is followed by a phase where the activities are reduced to a maintenance level. The multi-faceted intervention will be conducted in 2013/2014 and is described in detail in Rasmussen et al., 2013 [14]. According to Danish law, questionnaire based studies do not need approval by ethical and scientific committees, nor informed consent. However the intervention study received Ethics approval from the Committees on Biomedical Research Ethics of the Capital Region of Denmark, October 10th 2012, ref: H-4-2012-115 and is registered with a unique trial registration number: ISRCTN78113519.

### Workplace recruitment – adoption

Nurses’ aides in elderly care in Denmark are primarily employed in the municipalities. In Denmark there are 98 municipalities that vary greatly in size. We contacted the second largest municipality regarding participation in this intervention. The first contact with the municipality was established by contacting the director general of the health and care administration office by email and telephone. A meeting was subsequently arranged between working environment consultants from the municipality, working environment representatives from the employees as well as local union representatives. At the meeting, the aim, content and activities of the intervention aiming at reducing LBP were described in overall terms and the possibility of enrolment in the project was discussed. However, since it was a very costly project, the municipality wanted to secure financial aid to be able to run the project. In Denmark, workplaces can apply for a grant through the national Prevention Fund (established in 2007), which covers the cost of implementation of workplace interventions in order to reduce musculoskeletal disorders, impaired health and work ability and sickness absence and thereby prevent exclusion from the labor market [16]. The municipality applied for a grant in order to cover some of the expenses of participating in the intervention. A project description was prepared for the application and the municipality was granted 6.8 million DKK (approximately 900.000 €) for the implementation of the intervention. Only after receiving external funding, the intervention was initiated in the municipality.

After formal confirmation of collaboration the details about the recruitment of employees were settled. In this municipality, the administration of elderly care is divided into 9 districts (under the department of health). The researchers presented the study at a meeting for the managers of the 9 districts. Moreover they were given a short written description of the aim, content and activities of the project and possible benefits from participating in the study. Afterwards, they were given the opportunity to discuss the project with their employees and decide whether or not their district wanted to participate in the project.

### Participant recruitment – reach

Eligible participants were nurses’ aides employed in elderly care more than 20 hours a week and being 18–65 years of age. The primary reason for not including workers working less than 20 hours pr. week was due to the nature of the intervention. Workers working part time are more likely not to be able to participate in the intervention during their working time and will have to use their spare time. The nurses’ aides (care workers) in the elderly care were employed either in nursing homes or in home care. For supporting implementation, participation was also offered to the kitchen and cleaning personnel as well as janitors (service workers) belonging to the participating teams. Thus, the eligible study population consisted of low-educated service- and blue-collar workers in elderly care. There are approximately 4350 employees in total in the municipality of which approximately 3000 are nurses’ aides. The exclusion criteria to the study were unwillingness to participate in the multi-faceted intervention, long term sick-listed or not being permanently employed.

In the fall of 2012, all employees in the adopting districts were invited to a short information meeting of 30 minutes’ duration providing information about the project. Prior to the information meeting, written information about the aim and activities was distributed to all employees in a short information brochure. Because of the team structure in the municipality, it was necessary to conduct several information meetings (approximately 40) in order to reach as many of the employees as possible. At these meetings the employees were given a short questionnaire in which they were asked to give their consent or not consent to participate in the intervention. If the employees were not present at the meeting, their supervisor was given an envelope with information about the project and the questionnaire to hand out to the employees later on and to encourage them to complete the questionnaire and send it back in a stamped and addressed envelope.

### Outcome measures - adoption

To explore the representativeness of the participating districts, characteristics of the adopting and non-adopting districts were investigated. This was done by e-mailing an electronic questionnaire to the district managers with questions regarding the managers’ characteristics (sex, seniority), organizational characteristics (abatement, staff reduction, turnover rate, new work tasks, management, demand for the service, regulatory or legal requirements), current activities (ongoing projects), attitudes towards workplace health promotion and prevention, and musculoskeletal problems at the workplace and attitude towards the project (requirements for participation, relevance and expectations) and involvement of employees in the decision regarding participation. The questionnaires were emailed to the nine managers shortly after their responses about consenting to participate/not participate in the project. All nine managers responded to the questionnaire. Finally information regarding number of centers, sickness absence and accidents at the workplace was collected through workplace registrations. These data allow for the analysis of differences between adopting and non-adopting districts. Data were analyzed qualitatively since there are only nine managers and therefore nine respondents. For this we pooled the questions into three categories and calculated percentages of the answers to the questions within each category for adopters and non-adopters. The three categories were:

1) Organizational stability

2) Management

3) Working environment

### Outcome measures - reach

First we assessed the payroll to find eligible participants for the study. From the workplace registrations we gathered information about age, sex, weekly working hours, job seniority (0–1 years, 2–10 years, >10 years), work type (employed in nursing homes or in homecare), work shift (day shift, night/evening shift), job group (care workers or service workers), district and educational level (unskilled, low skilled (<2 years of education), and high skilled (≥2 years of education)). A questionnaire was distributed to all eligible employees, and those responding (responders) were compared with those not responding (non-responders).

Among responders, comparisons were made between those consenting to participate in the intervention (consenters) and those not consenting to participate in the intervention (non-consenters). From the questionnaires, information were collected on height and weight (from this we calculated the body mass index (BMI)), ethnicity (in which country are you born? [Response categories were: Denmark or other country]), and smoking (do you smoke? [Response categories were: yes/no]). To obtain information on the participants’ leisure time physical activity (LTPA), Saltin and Grimby’s validated questionnaire was used [17]. The following question was posed: “Looking back over the past year, what would you say fits best with your spare time activity: (i) Almost totally physically inactive or lightly physically active for less than 2 hours per week (e.g. reading, television, cinema), (ii) Lightly physically active for 2–4 hours per week (e.g. walking, bicycling, easy gardening, easy gymnastics), (iii) Lightly physically active for more than 4 hours per week or more strenuously physically active for 2–4 hours per week (e.g. fast walking, bicycling i.e. overtaking others, heavy gardening, strenuous gymnastics causing sweating and losing your breath) (iv) More strenuous physical activity for more than 4 hours per week or regular heavy training and possibly competition several times per week”. For the analyses, we dichotomized the response categories (iii) and (iv) into active and the response categories (i) and (ii) into inactive. Information regarding musculoskeletal symptoms during the previous 3 months was obtained with a slightly modified Nordic Musculoskeletal Questionnaire [18]. The following question was posed: “On a scale from 0–10 what was your worst pain during the last 3 months?” for the pain intensity with 0 indicating no pain and 10 indicating the worst pain. The question was posed for the low back, then neck/shoulders and then knees. For the analyses, we dichotomized the answers into pain (>0 on the pain intensity scale) and no pain (0 on the pain intensity scale). We made a combined variable of pain indicating pain in 1 or more body part by collapsing the dichotomized variables for each body part. However, if information from one body part was missing we considered the variable missing. Self-rated health was measured with a question from a Danish translation of the Short Form 36 (SF-36) questionnaire [19]. The question was: “In general, how would you rate your health?” with the response categories “very good”, “good”, “fair”, “poor”, and “very poor”. For the analyses, the variable was dichotomized into good health (‘very good” and “good”) and reduced health (“fair”, “poor”, and “very poor”). Sickness absence was measured using one question: “How many workdays in total have you been sickness absent within the last 3 months?”[20], and for the analyses, dichotomized as sickness absence (>0 days) and no sickness absence (0 days). Perceived physical exertion was measured by the question: “How would you rate your physical exertion during your current work?” with the response options on a 0–10 likert scale where 0 = not strenuous and 10 = maximal strenuous [21]. For the analyses, we divided the responses into three categories (tertiles) (light (0–5), moderate (6–7) and strenuous (8–10)). Finally we asked about reasons for not consenting to participate in the intervention. These data allow for further analyses of differences between consenters and non-consenters for the intervention.

### Statistical analysis

When comparing responders with non-responders and consenters with non-consenters, Student's t-test was conducted for the continuous variable age. A contingency table with Fisher's exact test was used to test for differences in the dichotomized variables sex, work type, work shift, job group, weekly working hours, physical exertion during work, ethnicity, BMI, self-rated health, smoking, LTPA, musculoskeletal pain in low back and in one or more body part (low back, neck/shoulders, knee), and information and relevance of the project. The Pearson’s chi^2^ was used to test for differences in job seniority, district and educational level. Finally, we analyzed odds ratios (OR) for non-responding and OR for non-consenting with binary logistic regressions. We made 3 models: 1) a crude analysis, 2) a model with adjustments for age and sex and 3) a model with adjustments for age, sex and district. Data were analyzed using SPSS (version 20.0) statistical software and SAS (version 9.3).

## Results

### Workplace recruitment

Four of the nine districts in the elderly care administration of the municipality adopted the project (44%). Altogether there were 37 centers in the nine districts. Each district consisted of a number of centers (between 3 and 6). The average number of centers among the adopting and non-adopting districts was 3 and 4.4, respectively. The number of eligible employees in the four adopting districts varied from 134 to 352 employees. Both the adopters and non-adopters reported that they had received sufficient information about the project to make a decision regarding participation in the project.

### Adopters and non-adopters

Given that we only have questionnaire data for nine districts (n = 9), we report them qualitatively according to the three categories: 1) Organizational stability, 2) Management and 3) Working environment (see Additional file 1 for answers to the questionnaires).

There were some differences between adopters and non-adopters regarding the overall organizational stability. Non-adopting districts reported to have a more unstable organization. Non-adopting districts also reported abatement, staff reduction and turnover rate more frequently than the adopting districts. Their management was more recently replaced and to a higher degree compared to the adopting districts predicted that there would be organizational changes during the study period.

The management of the adopting and non-adopting districts had some similarity with respect to overall attitudes towards responsibility of promoting health. However, in terms of their understanding of the project, some differences were present. The managers of the non-adopting districts found participating in the project more demanding both regarding economy and time, compared to the adopting districts. The managers of the adopting districts to a higher degree than the non-adopting districts believed, that the project would solve their needs and reduce the sickness absence, increase wellbeing at work, or increase quality in work. Finally, in all the adopting districts the managers had involved employee representatives in the decision regarding participation whereas only 2 of the non-adopting districts had involved employee representatives in the decision regarding participation.

The working environment among the non-adopting and the adopting districts were similar in many ways. There were no overall differences regarding mean sickness absence days among the adopting (16 days) and non-adopting (17 days) districts. No overall differences were found for mean numbers of accidents previous year among the adopting (72 accidents) and non-adopting (68 accidents) districts. Both the adopting and non-adopting districts reported that they did not have great problems with musculoskeletal pain and that the employees did not often complain about pain. Moreover, musculoskeletal pain was not considered a cause of sickness absence neither for the adopting or the non-adopting districts. However, both the adopting and non-adopting districts had prevention of musculoskeletal pain as a priority. Nearly all districts (8 out of 9) had more than 2 ongoing projects. One of the non-adopting districts had no ongoing projects. When looking at the specific purposes of the ongoing projects, the most reported purpose for both the non-adopting and adopting districts involved psychosocial working environment and management development and to a lesser degree health promotion projects. None of the adopting districts had ongoing projects concerning the physical working environment whereas more than half of the non-adopting districts had ongoing projects concerning the physical working environment.

We also asked the managers of the non-adopting districts for the main reason for not adopting the project (data not shown). The reasons were mostly related to the organizational stability as many of them had experienced or anticipated restructurings within the near future.

### Reach

### Participant recruitment

**Figure 1:**
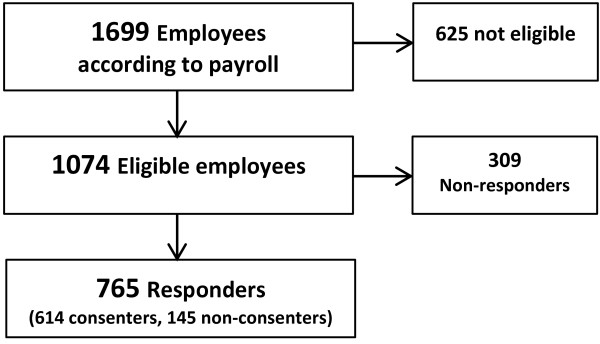
**The employee flow for the four adopting districts.** Out of 1699 employees there were 1074 eligible employees at the workplace. At the information meetings we established contact with 765 employees who filled out and returned the questionnaires (responders). Thus, there were 309 non-responders. From the responders 614 employees consented to participate and 145 employees were non-consenters.

In Figure 1 the flow of the eligible employees in the four adopting districts is shown. After assessing the payroll (n = 1699) for eligible participants we excluded 625 employees (37%) that were not eligible (not belonging to the job group, no longer employed, long term sick-listed or not being permanently employed). Out of the 1074 eligible employees there were 765 responders (71%). Approximately 20-25% responded independently from the information meetings. Among the responders, there were 614 consenters (57%). The reach percentage for responders in each district was 74% for district 1, 57% for district 2, 70% for district 3, and 71% for district 4 respectively. Equivalently, the reach percentage for consenters in each district was 57% for district 1, 57% for district 2, 64% for district 3 and 45% for district 4, respectively.

### Responders and non-responders

In Table 1 the differences between responders and non-responders are shown. Responders did not differ from the non-responders on age, sex, weekly working hours, work type and job group. There were fewer responders with low job seniority (0–1 years) and more responders with high seniority (>10 years) compared to non-responders. Also, there were fewer responders that are unskilled compared to the non-responders. There were fewer responders than non-responders from one of the districts - district 4. Finally, fewer responders worked evening/nightshift compared to non-responders.

**Table 1:** Characteristics of responders and non-responders

	**N**	**Responders**	**N**	**Non-responders**	**P-value**
		**(n = 765)**		**(n = 309)**	
***Demographic factors***					
**Age (years) (mean) (SD)**	765	47 (12)	309	47 (10)	NS
**Sex (% women)**	765	92	284	89	NS
***Work related factors***					
**Job seniority**	672		249		0.002
0-1 years (%)		13		20	
2-10 years (%)		45		49	
>10 years (%)		42		31	
**Weekly working hours**	676		247		0.000
>33 hours pr. week (%)		55		37	
31-33 hours pr. week (%)		15		15	
≤ 30 hours pr. week (%)		30		48	
**Work type (% nursing home)**	765	41	309	46	NS
**District**	765		309		0.000
District 1 (%)		31		27	
District 2 (%)		32		34	
District 3 (%)		27		21	
District 4 (%)		10		19	
**Job group (% engaged in care work)**	752	87	303	90	NS
**Educational level**	752		309		0.000
Unskilled (%)		9		16	
Low skilled (%)		27		27	
High skilled (%)		63		57	
**Work shift (% day shift)**	525	78	221	53	0,000

From workplace registrations responders were compared to non-responders on demographic factors and work related factors. n = number of responders, SD = standard deviation, NS = Non-Significant p>0.05.

The results of the logistic analyses are shown in Table 2. In the fully adjusted model, the odds for non-responding was significantly increased for workers with a job seniority of 2–10 years (OR = 1.70 (CI: 1.20-2.41)) and for workers with a job seniority of 0–1 years (OR = 2.32 (CI: 1.43-3.78)). Moreover working 30 hours pr. week or less increased the OR for non-responding to 4.15 (CI: 3.11-5.55). Workers who were unskilled also had increased OR for non-responding (OR = 4.14 (CI: 2.82-6.08)). Finally working evening/nightshift increased the OR for non-responding with 3.11 (CI: 2.18-4.42). Neither work type or work group influenced the OR for non-responding.

**Table 2:** Odds ratios for non-responding

	**Model 1**	**Model 2**	**Model 3**
	**OR (95% confidence interval)**	**OR (95% confidence interval)**	**OR (95% confidence interval)**
***Work related factors***			
**Job seniority**			
>10 years	REF	REF	REF
2-10 years	1.58 (1.17-2.15)	1.49 (1.07-2.08)	1.70 (1.20-2.41)
0-1 years	2.39 (1.61-3.54)	2.06 (1.30-3.25)	2.32 (1.43-3.78)
**Weekly working hours**			
>33 hours pr. week	REF	REF	REF
31-33 hours pr. week	1.24 (0.82-1.86)	1.23 (0.79-1.91)	1.39 (0.89-2.18)
≤ 30 hours pr. week	4.83 (3.71-6.29)	4.30 (3.25-5.70)	4.15 (3.11-5.55)
**Work type**			
Homecare	REF	REF	REF
Nursing homes	1.13 (0.89-1.44)	1.22 (0.95-1.58)	1.27 (0.97-1.66)
**District**			
District 1	4.24 (3.06-5.87)	3.98 (2.79-5.68)	-
District 2	1.75 (1.24-2.47)	1.77 (1.21-2.59)	-
District 3	REF	REF	-
District 4	4.12 (2.75-6.19)	5.07 (3.28-7.84)	-
**Job group**			
Care workers	REF	REF	REF
Service workers	1.37 (0.96-1.96)	1.17 (0.79-1.71)	1.45 (0.96-2.19)
**Educational level**			
High skilled	REF	REF	REF
Low skilled	0.87 (0.66-1.14)	0.97 (0.72-1.28)	0.98 (0.73-1.31)
Unskilled	6.29 (4.42-8.94)	4.89 (3.37-7.06)	4.14 (2.82-6.08)
**Work shift**			
Dayshift	REF	REF	REF
Evening/nightshift	2.08 (1.54-2.79)	2.62 (1.89-3.62)	3.11 (2.18-4.42)

From workplace registrations the odds ratios (OR) for non-responding was analyzed with binary logistic regressions. OR=odds ratios, REF=reference group.

Model 1: crude analysis, Model 2: adjusted for sex and age, Model 3: adjusted for sex, age and district.

### Consenters and non-consenters

There were no differences in demographic factors and BMI, smoking and leisure time physical activity between consenters and non-concenters (Table 3). With respect to health, more consenters reported reduced self-rated health, low back pain, and pain in 1 or more body parts compared to non-consenters. Consenters did not differ on job seniority, weekly working hours, district, job group, or sickness absence the last 3 months. For other work related factors there were fewer consenters working in nursing homes and fewer consenters that were unskilled and low skilled compared to non-consenters. Fewer consenters worked evening/nightshift compared to non-consenters. Moreover consenters reported a higher physical exertion during work compared to non-consenters. Finally, regarding project related factors, there was no significant difference in the percentage with sufficient information about the project, however nearly all the consenters (98%) found the project relevant compared to only 83% among non-consenters.

**Table 3:** Characteristics of consenters and non-consenters

	**N**	**Consenters**	**N**	**Non-consenters**	**P-value**
		**(n = 614)**		**(n = 145)**	
***Demographic factors***					
**Age (years) (mean)(SD)**	614	47 (10)	145	46 (11)	NS
**Sex (% women)**	614	92	145	90	NS
**Ethnicity (% Danish)**	609	86	140	90	NS
***Health***					
**BMI (% BMI > 25 (overweight and obese))**	544	46	107	45	NS
**Self-rated health (% reduced)**	609	33	139	24	0.020
**Smokers (% smokers)**	609	30	135	37	NS
**Leisure time physical activity (% inactive)**	606	84	135	89	NS
**Pain low back (% with pain)**	538	82	123	67	0.000
**Pain low back, neck, knee (% with pain in 1 or more body parts)**	440	92	106	78	0.000
***Work related factors***					
**Job seniority**	545		122		NS
0-1 years (%)		13		16	
2-10 years (%)		46		43	
>10 years (%)		42		42	
**Weekly working hours**	542		129		0.004
>33 hours pr. week (%)		56		50	
31-33 hours pr. week (%)		16		9	
≤ 30 hours pr. week (%)		28		41	
**Work type (% nursing home)**	614	41	145	46	0.046
**District**	614		145		NS
District 1 (%)		29		35	
District 2 (%)		32		33	
District 3 (%)		29		22	
District 4 (%)		10		10	
**Job group (% engaged in care work)**	614	87	145	83	NS
**Educational level**	614		145		0.007
Unskilled (%)		7		15	
Low skilled (%)		26		30	
High skilled (%)		65		55	
**Work shift (% day shift)**	419	80	101	70	0.034
**Physical exertion during work**	607		138		0.034
Light (%)		35		44	
Moderate (%)		40		41	
Strenuous (%)		25		15	
**Sickness absence previous 3 months (% yes)**	574	42	128	34	NS
***Project related factors***					
**Information about project (% with sufficient information)**	605	92	137	87	NS
**Relevance of the project (% that finds the project relevant)**	597	98	132	83	0.000

From workplace registrations consenters were compared to non-consenters on work related factors. From questionnaires consenters were compared to non-consenters on demographic factors, health and project related factors. n = number of responders, SD = standard deviation, NS = Non-Significant p>0.05, BMI= body mass index.

The results of the logistic analyses for non-consenting are shown in Table 4. Job seniority, job group, ethnicity, BMI, smoking, LTPA, and sickness absence did not influence the OR for non-consenting. In the fully adjusted model, working 30 hours or less pr. week increased the OR for non-consenting to 2.02 (CI: 1.08-3.79). Working evening/nightshift also increased the OR for non-consenting to 2.05 (CI: 1.09-3.83). With respect to health, having low back pain and having pain in one or more body parts decreased the OR for non-consenting to 0.48 (CI: 0.31-0.74) and 0.31 (CI: 0.17-0.56), respectively. Moreover perceiving the physical exertion during work as strenuous decreased the OR for non-consenting to 0.50 (CI: 0.29-0.87). Not having sufficient information about the project or not finding the project relevant increased the OR for non-consenting to 1.80 (CI: 1.02-3.17) and 8.44 (CI: 4.23-16.86), respectively. In the fully adjusted model, working in nursing homes also increased the OR for non-consenting with 2.43 (CI: 1.28-4.62). In model 2 adjusting for age and sex, being unskilled was associated with non-consenting with OR of 1.96 (CI: 1.05-3.66), however, after inclusion of district to the model, the association was no longer significant. Reduced self-rated health was only significant in the crude model with a decreased OR for non-consenting of 0.66 (CI: 0.44-0.99).

**Table 4:** Odds ratios for non-consenting

	**Model 1**	**Model 2**	**Model 3**
	**OR (95% confidence interval)**	**OR (95% confidence interval)**	**OR (95% confidence interval)**
***Work related factors***			
**Job seniority**			
>10 years	REF	REF	REF
2-10 years	0.91 (0.59-1.38)	0.86 (0.54-1.35)	1.03 (0.54-1.97)
0-1 Years	1.15 (0.64-2.07)	1.04 (0.54-2.01)	1.06 (0.38-2.94)
**Weekly working hours**			
>33 hours pr. week	REF	REF	REF
31-33 hours pr. week	0.56 (0.29-1.11)	0.57 (0.29-1.13)	0.99 (0.40-2.47)
≤ 30 hours pr. week	1.61 (1.07-2.42)	1.62 (1.07-2.45)	2.02 (1.08-3.79)
**Work type**			
Homecare	REF	REF	REF
Nursing homes	1.42 (0.96-2.11)	1.42 (0.95-2.11)	2.43 (1.28-4.62)
**District**			
District 1	1.52 (0.94-2.48)	1.52 (0.93-2.48)	-
District 2	1.33 (0.81-2.17)	1.33 (0.81-2.17)	-
District 3	REF	REF	-
District 4	1.33 (0.68-2.62)	1.29 (0.64-2.54)	-
**Job group**			
Care workers	REF	REF	REF
Service workers	0.67 (0.40-1.12)	0.70 (0.41-1.20)	0.69 (0.40-1.18)
**Educational level**			
High skilled	REF	REF	REF
Low skilled	0.77 (0.51-1.16)	0.80 (0.53-1.22)	1.10 (0.47-2.57)
Unskilled	1.87 (1.01-3.43)	1.96 (1.05-3.66)	2.33 (0.61-8.89)
**Work shift**			
Dayshift	REF	REF	REF
Evening/nightshift	1.64 (1.01-2.67)	1.65 (1.01-2.69)	2.05 (1.09-3.83)
***Demography***			
**Ethnicity**			
Born in Denmark	REF	REF	REF
Born in other country	0.65 (0.36-1.18)	0.67 (0.37-1.23)	0.67 (0.37-1.23)
***Health***			
**BMI**			
Normal weight	REF	REF	REF
Overweight (BMI > 25 (overweight and obese))	0.94 (0.62-1.43)	0.95 (0.62-1.46)	0.94 (0.61-1.44)
**Self-rated health**			
Good	REF	REF	REF
Reduced	0.66 (0.44-0.99)	0.67 (0.44-1.02)	0.66 (0.43-1.01)
**Smokers**			
Yes	REF	REF	REF
No	0.81 (0.56-1.19)	0.76 (0.51-1.12)	0.74 (0.50-1.10)
**Leisure time physical activity**			
Active	REF	REF	REF
Inactive	1.52 (0.88-2.62)	1.62 (0.91-2.89)	1.64 (0.92-2.92)
**Pain low back**			
No pain	REF	REF	REF
Pain	0.46 (0.30-0.70)	0.48 (0.31-0.74)	0.48 (0.31-0.74)
**Pain (low back, neck, knee)**			
No pain	REF	REF	REF
Pain	0.31 (0.18-0.54)	0.32 (0.18-0.57)	0.31 (0.17-0.56)
**Physical exertion during work (0-10)**			
Light (0-5)	REF	REF	REF
Moderate (6-7)	0.81 (0.55-1.20)	0.85 (0.57-1.28)	0.86 (0.57-1.29)
Strenuous (8-10)	0.49 (0.29-0.83)	0.51 (0.30-0.87)	0.50 (0.29-0.87)
**Sickness absence previous 3 months**			
No	REF	REF	REF
Yes	0.80 (0.54-1.18)	0.75 (0.50-1.11)	0.73 (0.49-1.09)
***Project related factors***			
**Sufficient information about project**			
Yes	REF	REF	REF
No	1.95 (1.13-3.37)	1.81 (1.03-3.20)	1.80 (1.02-3.17)
**Relevance of the project**			
Relevant	REF	REF	REF
Not relevant	8.21 (4.18-16.11)	8.35 (4.21-16.55)	8.44 (4.23-16.86)

From workplace registrations and questionnaires the odds ratios (OR) for non-consenting was analyzed with binary logistic regressions. OR=odds ratios, REF=reference group.

Model 1: crude analysis, Model 2: adjusted for sex and age, Model 3: adjusted for sex, age and district.

Among non-consenters who gave reasons for not participating, the most cited reason was lack of time, followed by not wanting the workplace to interfere with health. A few non-consenters also reported lack of knowledge on project content or no interest in the project (Table 5).

**Table 5:** Reasons for not participating among non-consenters

**Reasons for not participating**	**N**
The project is not interesting	12
I do not know what the project is about	14
My workplace should not interfere with my health	23
I have not got the time to participate	38
I do not wish to answer the question	53

N=number of non-consenters answering reasons for not participating for each of the questions. It was possible to answer yes to more than one question. The answers range from 12 to 53 answering yes to the specific question.

## Discussion

The main findings in this study showed that 44% of the eligible workplaces adopted the intervention. These workplaces had a more stable organization as well as a management with more positive beliefs of the intervention’s potential benefits. The reach was 71% for the questionnaire responders group and the reach for employees consenting to participate was 57%. Non-responders and non-consenters did not differ from the responders and consenters, respectively, on age, sex, and job group. More responders had high seniority, were working day shift, were working more than 30 hours pr. week, and were higher skilled compared to non-responders. The consenters and non-consenters showed no differences in BMI, smoking and leisure time physical activity, but consenters had more pain and reduced health, as well as higher physical exertion during work compared to non-consenters.

### Adoption

The 44 percent adopters in the present study is slightly lower than in other trials which have reported on adoption of health promoting initiatives in the workplace, and finding that 50-58% of the workplaces adopted the initiatives [22,23]. The municipality was granted financial aid to cover some of the expenses regarding implementation of the project but still some of the districts declined to participate. It therefore seems that the money was not a main incentive for participating.

In addition to investigating the percentage of adopters, we also investigated potential differences between adopters and non-adopters. For all 9 districts there is a policy regarding patient transfer techniques with mandatory courses for all new employees as well as brush-up courses every second year. When asking about additional projects nearly all districts (8 out of 9) had more than 2 ongoing projects. One of the non-adopting districts had no ongoing projects. The projects primarily focused on the psychosocial working environment and management development and to a lesser degree health promotion. However, none of the adopting districts had ongoing projects concerning the physical working environment whereas more than half of the non-adopting districts had ongoing projects concerning the physical working environment. This lack of existing projects on this particular topic in the adopting districts may explain the adoption of the present project focusing on the physical working environment to reduce LBP.

We found that the working environment reported by the managers was relatively similar between adopters and non-adopters, whereas there were differences in the management and organizational stability. The overall differences in management were primarily related to the managers’ assessment of the project requirements (time and expenses), with the managers of the non-adopting districts assessing them to be higher than the adopting districts. Also the managers of the non-adopting districts did not believe that the intervention would be beneficial in terms of solving their needs and reducing the sickness absence, increase wellbeing at work, or increase quality in work. Moreover, it was shown that non-adopters had a more unstable organization with more reporting abatement, staff reduction and turnover rate than the adopting districts. Their management was more recently replaced and they predicted that there would be organizational changes during the study period to a higher degree compared to the adopting districts. The reasons among non-adopters for not adopting the project were mostly related to the organizational stability as many of them had experienced or anticipated restructurings within the near future. These findings indicate that workplaces are more reluctant to adopt an initiative, if the organization is not stable. This corresponds well with the findings by Jørgensen et al. 2010 [22] reporting that cleaning workplaces facing organizational changes did not adopt the project. Moreover, the differences between the management among adopters and non-adopters indicate that management beliefs about the benefits of the project are important for adoption of the intervention. This is in accordance with previous studies [10]. Often it rests in the hands of a few individuals, usually senior managers, to decide whether or not a workplace will adopt a workplace program [24]. However, in this study all the managers of the adopting districts had involved employees in the decision regarding participation in contrast to the non-adopting districts. This may imply either that when a manager is convinced about the projects benefits, they are more likely to involve the employees in further decisions or that employee involvement increases the odds of adopting the initiative. In support of our findings on the importance of employee involvement Witte 1993 [25] reported that organizations with democratic management were more likely to adopt health promotions programs. However, we do not know whether employee involvement happened before or after the managers’ initial decision about participation. Future research is needed to more systematically examine these management factors and employee involvement and discover how they are linked to adoption of workplace health promotion programs.

### Reach of responders

In this study, 71% of the eligible population participated in an information meeting about the project (responders). Among the responders, most of them (80%) chose to participate, and thus information meetings seem to be an important recruitment tool. However, nearly 30% of the eligible employees did never attend an information meeting (non-responders). Particularly in one district (district 4), attendance at information meetings was lower compared to the other districts. District 4 was the smallest district with 134 eligible employees compared to up to 352 eligible employees in the other districts. Even though this district had fewer eligible employees, the relative reach of responders was lower. In this district, we offered fewer information meetings than in the other districts (4 compared to up to 21) due to a district management decision. Thus maybe higher accessibility of information meetings is important for the reach of participants as it has been suggested in a previous study [24]. Particularly some groups attended the information meetings less: evening and night shift workers (OR = 3.11), workers working 30 hours pr. week or less (OR = 4.15), newly employed workers (OR = 1.70-2.32) and workers with lower skill levels (OR = 4.15). Even though we had information meetings at all times of the day, many of the non-responders were employees working evening or night shift. Shift work has been suggested as a cause of unequal access to health promotion [26]. Therefore it seems that special initiatives are needed for reaching the group of employees working evening or night shift. The lower amount of responders working 30 hours or less pr. week is in accordance with previous studies showing that full-time workers are more likely to participate [5,27]. Among non-responders there was also a higher rate of newly employed workers. According to the authors’ knowledge, no studies on reach in worksite health promotion have focused on this aspect of newly employed workers. It has been suggested that for newly employed workers, the managers should be especially aware of their socialization process at the workplace [28] which may be of particular importance with respect to reach of newly employed workers in health promotion initiatives.

### Reach of consenters

Fifty-seven percent of the eligible employees consented to participate in the intervention. Reach corresponds well with previous studies, although there are large discrepancies ranging from 33% to 61% [5,9]. Robroek and colleagues [5] concluded that multiple component programs generally had higher participation rates, probably causing better reach to the target group. The multi-faceted intervention offered in the current study can therefore possibly explain the relatively high reach rate.

Among the consenters and non-consenters there were no differences in age, sex or health parameters such as BMI, smoking and leisure time physical activity. Other studies show contradicting results with respect to demographic factors such as age and sex between consenters and non-consenters [5]. Also for health-related determinants, there is no consistent evidence for a higher participation among healthier employees [5]. For example, studies have shown that obese employees are more likely to participate in workplace health promotion [5,22]. Equivalent to the reach of responders, workers working 30 hours or less pr. week to a higher degree did not consent to participate (OR = 2.02). Also working evening/nightshift increased the OR for non-consenting (OR = 2.05).

The multi-faceted intervention was designed to prevent and reduce LBP and consequences of LBP among nurses’ aides in elderly care in Denmark. This study shows that consenters had more pain and reduced self-rated health compared to non-consenters. This indicates that the study appealed to an unhealthier proportion of the nurses’ aides, which is consistent with the findings of a study by Jørgensen et al. 2010 on cleaners [22]. We also found that the consenters reported a higher physical exertion during work. In the current study, the intervention focused on preventing and reducing LBP, which included, among other things, a focus on reduction of physical exertion during work. This may explain why employees experiencing high physical exertion during work were actually motivated to enroll in the study. The reach of employees with health-issues in this study is highly relevant for the workplace health promotion and work environment intervention aiming at reducing LBP. However, since this is a job group with high prevalence of LBP [29,30] a high reach of healthy employees is less likely to occur. In a preventative perspective, reach of the smaller proportion of healthy employees is also relevant, but it may require a different recruitment strategy.

Like non-responders, non-consenters were more likely to work evening or night shift and have lower skill levels. When investigating reasons for not participating, 11% of the evening/night shift workers answered lack of time, whereas for day shift workers the number was 3% to the same question. However, reasons for the lower reach of evening and night shift workers as well as lower skilled workers need future studies, to understand why certain groups choose to participate less. Furthermore, future workplace initiatives should pay attention to this issue, since particularly high risk groups may miss out on important health promotion opportunities.

Nearly all the consenters (98%) found the project relevant in comparison to 83% of the non-consenters. However, the odds for not consenting to participate were more than 8 for those finding the project not relevant compared to those finding the project relevant. The high percentage of the non-consenters still finding the project relevant points towards other reasons for not participating. The non-consenters, when asked about the reasons for not consenting to participate, answered: *lack of time*, *not wanting the workplace to interfere with health*, *not knowing what the project is about*, and *not finding the project interesting*. A study by Robroek and colleagues [31] on moral issues in workplace health promotion shows similar results: The main reasons for non-participation was *lack of time* and *not wanting the workplace to interfere with health*. We would have assumed that the reason *lack of time* was absent in the current study, since participation was offered during paid working hours.

The recruitment process of both workplaces and employees of this study provides knowledge of the representativeness of the population, and consequently the external validity and the generalizability of the results of the intervention study. Using the RE-AIM framework’s two measures, reach and adoption, we gain important insights on the representativeness of the study, which may improve the ability of practitioners, workplaces and researchers to successfully plan and implement future workplace health promotion and work environment interventions.

### Strengths and limitations

The focus on both the individual and the organizational level is a strength of the study. Another strength of the study is the collection of data on adopters and non-adopters, so that we can report on characteristics and not just the percentage of adopters. Our measure of reach on both responders and concenters level, is a strength as it besides information about those who actually did participate in the intervention also provides knowledge of the absolute number, proportion, and representativeness of individuals who are willing to participate in a given intervention. Moreover the large amount and relevance of information available for comparing responders and non-responders as well as consenters and non-consenters offers an opportunity to investigate the feasibility and external validity of the intervention and intervention effects.

A limitation of the study is that only one municipality was represented in this study, meaning that this sample of nurses’ aides is not a representative sample of nurses’ aides in Denmark. Furthermore, this time-consuming intervention was provided to the employees with a low monetary cost to the workplace (as they were granted money for the implementation of the intervention). Thus, we are unable to predict participation in future similar workplace interventions in which a greater investment of resources is required from the workplace.

## Conclusion

For the multi-faceted workplace health promotion and work environment intervention among nurses’ aides in elderly care, there was a satisfactory adoption of 44% and a high reach of 71% for the questionnaire responders and a reach for consenting to participate of 57%. We found differences in the management and organizational stability with workplaces adopting this intervention having a stable organization as well as a management with positive beliefs of the intervention’s potential benefits compared to non-adopters. Our findings suggest that our recruitment efforts yielded a population that was representative of the target population of nurses’ aides with respect to demographic factors, and health. We succeeded in reaching a majority of employees with musculoskeletal pain which is the main focus of the intervention. However, future initiatives particularly aiming at prevention may consider specially targeted efforts to attract employees without pain. Moreover, non-responders and non-consenters were more likely to work evening or night shift, working less than 30 hours pr. week and have lower skill levels, and among non-responders there were also a higher rate of newly employed workers. Specific recruitment efforts may be needed to reach lower skilled, part time workers, newly employed and evening and nightshift workers.

## Competing interests

The authors declare that they have no competing interests.

## Authors’ contributions

CDNR led the writing of the manuscript, and wrote the first draft of the manuscript. CDNR, MBJ, AHO and KS contributed to the design of the study and all authors contributed to the study protocol. AKL collected and structured the adoption data and all authors contributed to the statistical tests run by CDNR. All authors read and approved the manuscript.

## Pre-publication history

The pre-publication history for this paper can be accessed here:

http://www.biomedcentral.com/1471-2288/14/60/prepub

## Supplementary Material

Additional file 1 Adoption data. Click here for file
