# Supplementary material for: Adoption of workplaces and reach of employees for a multi-faceted intervention targeting low back pain among nurses’ aides
Source: BMC Med Res Methodol. 2014 May 1;14:60. doi: 10.1186/1471-2288-14-60 (PMC4021388; doi:10.1186/1471-2288-14-60)
Supplement: Additional file 1 — Adoption data. [file 1471-2288-14-60-S1.docx]

**Additional file 1: Adoption data**

Responses from questionnaires sent to the nine district managers.

|  | **Adopting districts (n=4)** | **Non-adopting districts (n=5)** |
| --- | --- | --- |
| Have you had sufficient information about the project to make a decision regarding participation in the project? |  |  |
| Yes (numbers, %) | 4 (100%) | 5 (100%) |

| **Organizational stability** | **Adopting districts (n=4)** | **Non-adopting districts (n=5)** |
| --- | --- | --- |
| Abatement – yes (numbers, %) | 3 (75%) | 4 (80%) |
| Staff reduction – yes (numbers, %) | 3 (75%) | 5 (100%) |
| High turnover rate of employees – yes (numbers, %) | 1 (25%) | 2 (40%) |
| Reorganization – yes (numbers, %) | 4 (100%) | 5 (100%) |
| New work tasks – yes (numbers, %) | 4 (100%) | 5 (100%) |
| New management – yes (numbers, %) | 3 (75%) | 5 (100%) |
| Increased demand for services – yes (numbers, %) | 1 (25%) | 1 (20%) |
| New regulatory or legal requirements – yes (numbers, %) | 1 (75%) | 3 (60%) |
| It is very likely that organizational changes will happen during the project  Yes (%)  Partly (%)  No (%)  Don’t know (%) | 25  75  0  0 | 60  20  20  0 |

| **Management** | **Adopting districts (n=4)** | **Non-adopting districts (n=5)** |
| --- | --- | --- |
| Sex of the manager– Female (numbers, %) | 4 (100%) | 2 (40%) |
| Seniority of the manager (mean years) (min, max) | 6 (1, 15) | 10 (1, 27) |
| Are employee representatives involved in the decision regarding participation in the project?  Yes (%)  Partly (%)  No (%) | 100  0  0 | 40  40  20 |
| The workplace has a responsibility for promoting health among the employees  Yes (%)  Partly (%)  No (%)  Don’t know (%) | 75  25  0  0 | 80  20  0  0 |
| Maintaining healthy is the employees own responsibility  Yes (%)  Partly (%)  No (%)  Don’t know (%) | 50  50  0  0 | 20  80  0  0 |
| The requirements for participating in the project is demanding, there are considerable economic costs associated with participation in the project, the project is time consuming for the management, the project is time consuming for the employees  Yes (%)  Partly (%)  No (%)  Don’t know (%) | 31.3  43.7  25  0 | 60  20  15  5 |
| The project will solve the needs that we have at this workplace  Yes (%)  Partly (%)  No (%)  Don’t know (%) | 75  25  0  0 | 20  40  20  20 |
| It is very likely that the project will reduce the sickness absence, increase wellbeing at work, or increase quality in work  Yes (%)  Partly (%)  No (%)  Don’t know (%) | 58  42  0  0 | 7  47  20  27 |

| **Working environment** | **Adopting districts (n=4)** | **Non-adopting districts (n=5)** |
| --- | --- | --- |
| Number of centers (mean number) (min, max) | 3 (3, 5) | 4.4 (3, 6) |
| Employee sickness absence (mean days the previous year) (min, max) | 16 (13, 19) | 17 (15, 19) |
| Employee accidents (mean number the previous year) (min, max) | 72 (47, 95) | 68 (35, 95) |
| Ongoing projects  None (%)  1 project (%)  2 projects (%)  More than 2 projects (%) | 0  0  0  100 | 20  0  0  80 |
| Purpose of ongoing projects  Psychosocial working environment (%)  Management development (%)  Physical working environment (%)  Health promotion (%) | 50  50  0  25 | 80  80  60  40 |
| Employees participate to a large extent in health and prevention projects  Yes (%)  Partly (%)  No (%)  Don’t know (%) | 0  100  0  0 | 0  80  0  20 |
| Musculoskeletal pain is a problem at this workplace, employees often complain about pain, pain is the primary cause of sickness absence  Yes (%)  Partly (%)  No (%)  Don’t know (%) | 0  25  42  33 | 0  20  53  27 |
| Prevention of musculoskeletal pain is a priority at this workplace  Yes (%)  Partly (%)  No (%)  Don’t know (%) | 50  50  0  0 | 40  40  20  0 |
